# A Comparative Evaluation of Post-operative Pain Management Using Erector Spinae Plane Block and Oblique Transverse Abdominis Plane Block in Patients Undergoing Laparoscopic Cholecystectomy

**DOI:** 10.7759/cureus.35750

**Published:** 2023-03-04

**Authors:** Vaddamanu Mounika, Lingaraj Sahu, Krishna Mishra, Partha S Mohapatra

**Affiliations:** 1 Department of Anesthesiology, Kalinga Institute of Medical Sciences, Bhubaneswar, IND; 2 Department of Community Medicine, Kalinga Institute of Medical Sciences, Bhubaneswar, IND

**Keywords:** nerve block, ultrasound-guided, ultrasound, erector spinae, oblique subcostal transverse abdominis plane, laparoscopic cholecystectomy

## Abstract

Introduction

Acute pain following laparoscopic surgeries interferes with the rehabilitation of the patient. Knowledge about the pain pathway from a particular area helps in blocking pain transmission at different sites. Ultrasonography (USG)-guided peripheral nerve blocks help in controlling pain better than non-steroidal anti-inflammatory drugs (NSAIDS) and opioids since they directly act by interrupting the pain pathway and interfere less with the physiology of the body. This study was planned with the objectives to evaluate the analgesic efficacy of USG-guided erector spinae plane block (ESPB) and oblique subcostal transversus abdominis plane (OSTAP) block in patients undergoing elective laparoscopic cholecystectomy, to compare the analgesic requirements in both groups, and to compare the VAS scores in both groups.

Materials and methods

A total of 138 patients were randomized into two groups, with 69 patients in each group, and received either bilateral ESP (group ‘E’) or bilateral OSTAP (group ‘O’). Those in group E received the block at the T7 level, and those in group O received the block in the subcostal region, with 20 ml of 0.2% ropivacaine and 4 mg of dexamethasone. The procedures were done after securing the airway. The total analgesic requirement and visual analogue scale (VAS) scoring in the first 24 hours post-operatively were observed, along with intra-operative opioid consumption. The opioid requirement, block-related complications, and patient feedback regarding post-operative pain control were also assessed. The results were analyzed using the Statistical Package for the Social Sciences (SPSS) software version 23.0 (IBM Corp., Armonk, NY). Continuous and categorical data were analyzed using appropriate statistical analysis. A p-value <0.05 was considered statistically significant.

Results

The VAS scores were significantly lower during the first post-operative day in group E than in group O. In group E, VAS scoring was less than 4 for the first 24 hours post-surgery. In group O, VAS scores remained greater than or equal to 4 after four hours post-surgery, thereby indicating that the patients required opioids. Only seven patients in group E received tramadol, compared to 62 patients in group O. The mean tramadol requirement of seven patients in group ‘E’ was 65.71 ± 26.3 mg, and the mean tramadol requirement of 62 patients was 114.56 ± 36.8 mg (p = 0.0012). The patients in group ‘O’ demanded tramadol significantly more times than those in group ‘E’.

Conclusion

It was concluded that USG-guided ESP block provides better pain control and decreases consumption of opioids post-operatively than OSTAP block in patients undergoing laparoscopic cholecystectomy surgeries. The block was found to be safe with no adverse effects, therefore it can be a part of multimodal analgesia.

## Introduction

Pain control is one of the important roles of anesthesiologists. Modern-day anaesthesia practise extends beyond the operation theatre (OT). The anaesthesiologist plays a major role in relieving pain post-operatively, which can be done in modern-day practise with the help of peripheral nerve blocks. Accessibility to ultrasound in the OT has made these procedures relatively hassle-free. Peripheral nerve blocks have better pain control effects than non-steroidal anti-inflammatory drugs (NSAIDS) and opioids. Knowledge about the pain generator and anatomical knowledge of the nerves that conduct pain from a particular area help block the pain pathway at different sites. Pain is defined by the IASP (International Association for the Study of Pain) as "an unpleasant sensory and emotional experience associated with, or resembling that associated with, actual or potential tissue damage [1]. Surgical techniques cause tissue damage and, hence, cause pain. Though laparoscopic surgeries are minimally invasive, patients may develop acute pain postoperatively, which might interfere with rehabilitation. Pain perception varies from person to person; in patients with a low pain threshold, it is difficult to manage with opioids and NSAIDs even at the maximum dose. Besides this, the opioids and NSAIDs cause delayed recovery, gastritis, nausea, and vomiting, making the patient more intolerant. Opioids are the main step of intra-operative and post-operative pain management. Ultrasound-guided transverse abdominis plane block (TAPB), oblique subcostal transverse abdominis plane block (OSTAPB), and erector spinae plane block (ESPB) effectively reduce the consumption of opioids and NSAIDs both in the intra-operative and post-operative periods [2-4]. Ultrasound-guided peripheral nerve block usage is increasing in popularity because it causes less interference with the physiology of the body as they act by interrupting the pain pathway, which can be assessed by using the visual analogue scale (VAS). Published studies reveal that TAPB, OSTAPB, and ESPB block effectively, thereby decreasing the post-operative pain in laparoscopic cholecystectomy [5,6], but there is a paucity of literature on the comparative evaluation between the blocks to find out the superiority among them to manage the post-operative pain in laparoscopic cholecystectomy. Hence, the present study was planned as a trial between OSTAPB and ESPB with the objectives to evaluate the analgesic efficacy of ultrasound-guided ESPB and OSTAPB in patients undergoing laparoscopic cholecystectomy, compare the analgesic requirements in both groups and compare the VAS scores in both groups.

## Materials and methods

The present study was a prospective randomized trial conducted in a tertiary care centre in India. The study was carried out after getting the institutional ethical committee's approval (KIIT/KIMS/IEC/413/2020). A total of 138 patients satisfying the inclusion and exclusion criteria were enrolled in the study. The sample size was calculated to be 69 for each group, considering the effect size of 0.478, the alpha error of 5%, and the power (1-beta) of 80%. Randomization was done using a computer-generated random number table. The patients in both groups were induced and the airways were secured. Then they received either bilateral ESP (group ‘E’) at T7 level or bilateral OSTAP (group ‘O’) with 20 ml of 0.2% ropivacaine and 4 mg dexamethasone. All patients belonging to the American Society of Anesthesiologists (ASA) I and II, including both males and females in the age group between 18 and 70 years, were included in the study. Patients with known allergies to the drugs under study, infections at the site of injection, and patients with coagulopathies were excluded from the study.

After recording the baseline vitals, the patients were pre-medicated with an injection of glycopyrrolate (0.2 mg) and midazolam (1 mg) intravenously (I.V.). Induction of anaesthesia was done with 2-3 mg/kg propofol, nalbuphine 0.1 mg/kg, and vecuronium 0.1 mg/kg, followed by tracheal intubation, and a secured airway was maintained. Nalbuphine was chosen as there is a paucity of supply of fentanyl and morphine, and hence the revised institutional guidelines were followed for the process. Standard monitors like an electrocardiogram (ECG), non-invasive blood pressure (NIBP), and pulse oximeter to record SpO_2_ were attached to the patient. Following this, according to group allocation, either a bilateral ESP or bilateral OSTAP block was performed. Anaesthesia was maintained with O_2_ and N_2_O in a ratio of 1:2, along with isoflurane at 0.8-1% in the vaporizer. A 12 mmHg intraperitoneal pressure was created with carbon dioxide (CO_2_) insufflation for the laparoscopy procedure.

Patients in group E received an ESPB block in the lateral position. The T7 vertebrae were identified by counting both from above the T1 vertebra and below the T12 vertebra using USG. Following confirmation of the lateral end of the transverse process, the trapezius, rhomboid major, and erector spinae muscles were visualized just above the transverse process. Following strict aseptic precautions, needling with a stimuplex needle was done by in-plane technique in the cephalo-caudal direction. The needle tip was placed at the sub-erector spinae plane. Hydrodissection was done with 1 ml of normal saline. After satisfactory dissection of the sub-erector spinae plane, 20 ml of 0.2% ropivacaine and 1 ml (4 mg) of dexamethasone were deposited. The same procedure was repeated on the other side.

Patients in group O received OSTAPB in the supine position. The probe was placed in an oblique plane below the lower costal margin. The rectus abdominis muscle with its posterior rectus sheath and the transverse abdominis muscle were identified. Following strict aseptic precautions, an echogenic stimuplex needle was placed using an in-plane technique until the tip of the needle reached the fascia between the rectus abdominis and the transverse abdominis muscles. Hydrodissection was done with 1 ml of normal saline. After satisfactory dissection of the transverse abdominis, the same steps as for the other group were followed.

At the time of incision, pneumoperitoneum, and gall bladder fossa dissection, if parameters were found to be 20% higher than the baseline, an additional dose of 0.1 mg/kg injection Nalbuphine was given IV. Patients were monitored in post-operative period for pain using VAS. Rescue analgesia was given in the form of systemic analgesia. Inj. tramadol was given at 1 mg/kg with gradually increasing doses of 0.5 mg/kg up to 2 mg/kg if VAS>4 and the time was noted. Injection Diclofenac sodium 1 mg/kg was given by slow IV if the pain was not controlled with tramadol. Tramadol was kept as a rescue analgesic in the current study and was given at the patient's demand. Patients were monitored for any adverse effects like nausea, vomiting, or shivering. All the patients in either group received injections Paracetamol 1 gm IV immediately after surgery, and the second dose was given after 12 hours. As multimodal analgesia was given, paracetamol was given at an interval of 12 hours instead of an eight-hourly dose.

After the patients were discharged from the hospital, feedback was collected telephonically. The extent of satisfaction with the post-operative pain control was measured with the 11-point numeric pain rating scale (0-10), with zero being the worst pain experience and 10 being no pain [7].

Data were analyzed using the IBM SPSS software version 23. For a continuous variable, the data were presented as median + IQR (interquartile range), and the categorical variables were presented as percentages. A p-value ≤ 0.05 was considered statistically significant in the present study.

## Results

The present study evaluated the analgesic efficacy of two blocks, namely ESPB and OSTAB. Table 1 depicts the comparison of both groups in various aspects.

**Table 1:** Comparison of demographic data, ASA status, height, weight and duration of the surgery between the groups ASA: American Society of Anesthesiologists

Variables	Group ‘E’	Group ‘O’	p-value
Gender			
Males	33 (47.8%)	25 (36.2%)	0.168
Females	36 (52.2%)	44 (63.8%)	
ASA status			
I	48 (69.56%)	41 (59.42%)	0.213
II	21 (30.44%)	28 (40.58%)	
	Mean ± IQR		
Age (years)	40 (31–55)	50 (40–57)	0.018
Weight in kg	67 (59–75)	64 (56–70.5)	0.125
Height in cm	160 (153–165)	155 (150–160)	0.099
Duration of surgery in min	120 (90–120)	100 (90–120)	0.116

In the above table, it is seen that the median age ± IQR was significantly lower in group ‘E’ than in group ‘O’. This was also found to be statistically significant. Other parameters like gender, ASA status, height, weight, and duration of the surgery were comparable between both groups. The variation of intra-operative vital parameters was found to be comparable in both groups without any statistically significant difference. The intra-operative hemodynamic parameters at the skin incision, at the creation of the pneumo-peritoneum, and during gall bladder fossa dissection in comparison to baseline parameters revealed no significant change in either group in comparison to baseline (not more than a 20% increase from the baseline). Table 2 represents the comparison of VAS scores between the groups.

**Table 2:** Comparison of VAS score between the groups VAS: Visual Analogue Scale

Time (in hours after surgery)	VAS scoring		p-Value
	Group ‘E’ (median ± IQR)	Group ‘O’ (median ±IQR)	
0–4	1.0 (1.0–2.0)	2.0 (2.0–2.0)	<0.001
4–8	3.0 (2.0–3.0)	4.0 (3.0–4.0)	<0.001
8–12	3.0 (2.0–3.0)	3.0 (3.0–4.0)	<0.001
12–18	3.0 (2.0–3.0)	4.0 (3.0–5.0)	<0.001
18–24	3.0 (2.0–3.0)	3.0 (3.0–4.0)	<0.001

The VAS score was less than 4 until four hours post-surgery in both groups. After that, the VAS remained significantly high in group O with a median value of VAS ≥ 3 as compared to the other group. This was found to be statistically significant. The findings were similar with a higher VAS score in group O up to 18-24 hours post-surgery. Table 3 depicts the comparison of postoperative opioid requirements between the groups.

**Table 3:** Comparison of postoperative opioid requirement between the groups FDT: first dose of tramadol in hours after surgery, NDT: number of doses of tramadol required

Variables	Group		p-Value
	E n(%)	O n(%)	
FDT			
0–4	0 (0.0%)	0 (0.0%)	<0.001
4–8	1 (1.4%)	37 (53.62%)	
8–12	2 (2.9%)	17 (24.6%)	
12–18	4 (5.8%)	3 (4.3%)	
Not required till 24 hours	62 (89.9%)	12 (17.3%)	
NDT (n)			
0	62 (89.9%)	12 (17.4%)	<0.001
1	6 (8.7%)	6 (8.6%)	
2	1 (1.4%)	35 (50.7%)	
3	0 (0.0%)	11 (15.9%)	
4	0 (0.0%)	5 (7.2%)	
Mean ± SD	65.71±26.3	114.56 ± 36.8	0.0012

As depicted in Table 3, around 89.9% of the patients in group E did not have an opioid requirement up to 24 hours following surgery, thereby depicting that the ESP block is more efficacious with respect to analgesic effect than the OSTAP block. This difference between the groups was found to be statistically significant. Similar results were also observed at different durations following surgery. There was significantly less requirement for tramadol in group ‘E’ in comparison to group ‘O’ (p=0.0012). None of the patients from both groups demanded for the second line of analgesics (i.e., Inj. diclofenac sodium).

The patient satisfaction score as per the feedback was significantly higher in group E [median ± IQR- 7.0 (7.0-7.0)] than in group O [median ± IQR- 4.0 (3.0-4.0)]. This difference in score was also found to be statistically significant (p≤0.001). It was found that in group E none of the patients reported nausea and vomiting in the post-operative period, while it was reported among 9 (13.04%) of the patients in group O. This difference was also found to be statistically significant (p=0.0058).

## Discussion

In the present study, patients from group E had better pain control during the first 24 hours following surgery. The first analgesic demand of this group of patients was significantly delayed compared to that of group O. The VAS and the total tramadol requirement in patients from group E were significantly less. The patients in this group also demanded analgesics less frequently than the patients in group O. The incidence of post-operative nausea and vomiting (PONV) was significantly lower in group E patients. The patient satisfaction score for post-operative pain control was higher in patients in group E than in group O. In a case report by Petsas et al., who performed a bilateral ultrasound-guided ESP block at the T6 level scheduled for laparoscopic cholecystectomy, it was reported that there was no analgesic requirement up to 10 hours post-operatively [8]. This finding is similar to the findings of the present study. Similar results are reported by Altiparmak et al. [9]. Daghmour et al. conducted a meta-analysis study on bilateral erector spinae plane block for post-operative analgesia in laparoscopic cholecystectomy, which showed a significant reduction in post-operative intravenous opioid consumption reported up to 24 hours after surgery. The study concluded that the block was effective in terms of reducing post-operative opioid consumption and the time required for the first rescue analgesic [10].

In another study conducted by Routray et al., it was concluded that rescue analgesic paracetamol consumption was lower in the ESPB group and the time to the first rescue analgesia request was longer in the ESPB group. This difference was found to be statistically significant; the results of which are persistent to the findings of the present study [11]. In a study conducted by Ozdemir et al., it was concluded that intra-operative and postoperative fentanyl requirements were lower in ESPB and the time to first rescue analgesic need was longer in ESPB. The numerical rating scale (NRS) scores were also lower in ESPB in comparison to OSTAPB [12]. Sahu et al. in their RCT compared the efficacy of ESPB and OSTAPB at T7 but did not find any significant difference in intraoperative opioid requirement between the groups, whereas there was a statistically significant difference in the mean VAS between the groups, which remained significantly lower in the ESP group during the first 24 hours as compared to OSTAPB. They concluded that ESPB was a superior block for laparoscopic cholecystectomy in comparison to OSTAPB [13]. In the present study, the patients in group O demanded tramadol significantly more times than those in group E, and the first analgesic demand was significantly delayed in group E (p < 0.001). These results are similar to the above three studies [11-13]. Shahid et al. conducted a study comparing the effects of paracetamol and tramadol on postoperative pain and found that the use of tramadol significantly increases the incidence of PONV. Similar to this, in the present study, nine patients (13.0%) from group ‘O’ whose tramadol requirement was higher developed PONV. None from group ‘E’ had PONV [14]. In the present study, feedback regarding post-operative pain relief was collected after the patient's discharge from the hospital. Similar to Sahu et al. significantly more patients from the ESPB group were satisfied in comparison to the OSTAP group [13].

The present study had some limitations, as the blocks were performed at two different sites and the needle prick site was covered with sterile dressing pads; hence, the blinding could not be performed during the procedure. The perception of pain varies from patient to patient being subjective. In conscious patients, different scales are available that can be used to measure the pain for better measurement, but this scale cannot be used during the intra-operative period as the patients were under general anesthesia, hence the change in hemodynamic parameters was considered to measure the intra-operative pain. There were other confounders that could mask this effect. As these blocks were performed after induction, the extent of the dermatome block could not be assessed.

## Conclusions

Based on the findings of the present study, it was concluded that USG-guided ESPB is more efficacious than OSTAPB and provides better pain control, thereby decreasing the consumption of opioids in the post-operative period compared with OSTAPB in patients undergoing laparoscopic cholecystectomy surgeries. The block was found to be safe, with no reported adverse effects among the study participants. The analgesic demand was significantly delayed in patients who received ESPB compared with those who received OSTAPB. The patient satisfaction score for post-operative pain control was higher for the ESPB group. This block may be considered an effective one for laparoscopic cholecystectomy. Other RCTs should be undertaken in order to explore the other possible benefits of this block in various other surgical procedures.
